# Generalised monogamy relation of convex-roof extended negativity in multi-level systems

**DOI:** 10.1038/srep36700

**Published:** 2016-11-18

**Authors:** Tian Tian, Yu Luo, Yongming Li

**Affiliations:** 1 College of Computer Science, Shaanxi Normal University, Xi’an, 710062, China

## Abstract

In this paper, we investigate the generalised monogamy inequalities of convex-roof extended negativity (CREN) in multi-level systems. The generalised monogamy inequalities provide the upper and lower bounds of bipartite entanglement, which are obtained by using CREN and the CREN of assistance (CRENOA). Furthermore, we show that the CREN of multi-qubit pure states satisfies some monogamy relations. Additionally, we test the generalised monogamy inequalities for qudits by considering the partially coherent superposition of a generalised W-class state in a vacuum and show that the generalised monogamy inequalities are satisfied in this case as well.

Quantum entanglement is one of the most important physical resources in quantum information processing^1^,^2^,^3^,^4^. As distinguished from classical correlations, quantum entanglement cannot be freely shared among many objects. We call this important phenomenon of quantum entanglement monogamy^5^,^6^. The property of monogamy may be as fundamental as the no-cloning theorem^7^, which gives rise to structures of entanglement in multipartite settings^8^,^9^. Some monogamy inequalities have been studied to apply entanglement to more useful quantum information processing. The property of monogamy property has been considered in many areas of physics: it can be used to extract an estimate of the quantity of information about a secret key captured by an eavesdropper in quantum cryptography^10^,^11^, as well as the frustration effects observed in condensed matter physics^12^,^13^ and even black-hole physics^14^,^15^.

The monogamy relation of entanglement is a way to characterise different types of entanglement distribution. The first monogamy relation was named the Coffman-Kundu-Wootters (CKW) inequality^8^. The monogamy property can be interpreted as the following statement: the amount of entanglement between *A* and *B* plus the amount of entanglement between *A* and *C* cannot be greater than the amount of entanglement between *A* and the *BC* pair. Osborne and Verstraete later proved that the CKW inequality also holds in an *n*-qubit system^9^. Other types of monogamy relations for entanglement were also proposed. Studies have found that the monogamy inequality holds in terms of some entanglement measures, negativity^16^, squared CREN^17^, entanglement of formation^18^,^19^,^20^, Rényi entropy^21^ and Tsallis entropy^22^,^23^. The monogamy property of other physical resources, such as discord and steering^24^, has also been discussed. There can be several inequivalent types of entanglement among the subsystems in multipartite quantum systems, and the amount of different types of entanglement might not be directly comparable to one another. Regula *et al*. studied multi-party quantum entanglement and found that there was strong monogamy^25^. Additionally, generalised monogamy relations of concurrence for N-qubit systems were also proposed by Zhu *et al*.^26^.

In this paper, we study the generalised monogamy inequalities of CREN in multi-qubit systems. We first recall some basic concepts of entanglement measures. Then, monogamy inequalities are given by the concurrence and negativity of the *n*-qubit entanglement. Furthermore, we consider some states in a higher-dimensional quantum system and find that the generalised monogamy inequalities also hold for these states. We specifically test the generalised monogamy inequalities for qudits by considering the partially coherent superposition of a generalised W-class state in a vacuum, and we show that the generalised monogamy inequalities are satisfied in this case as well. These relations also give rise to a type of trade-off in inequalities that is related to the upper and lower bounds of CRENOA. It shows the bipartite entanglement between *AB* and the other qubits: especially under partition *AB*, a two-qubit system is different from the previous monogamy inequality that is typically used.

## Results

This paper is organised as follows: in the first subsection, we recall some basic concepts of concurrence and negativity. We present the monogamy relations of concurrence and negativity in the second subsection. In the third subsection, the generalised monogamy inequalities of CREN are given. The fourth subsection includes some examples that verify these results.

### Preliminaries: concurrence and negativity

For any bipartite pure state |*ψ*〉_*AB*_ in a *d* ⊗ *d*′ (*d* ≤ *d*′) quantum system with its Schmidt decomposition,


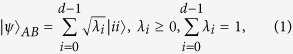


the concurrence 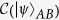 is defined as^27^


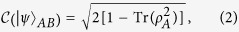


where *ρ*_*A*_ = *tr*_*B*_ (|*ψ*〉_*AB*_〈*ψ*|). For any mixed state *ρ*_*AB*_, its concurrence is defined as


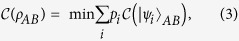


where the minimum is taken over all possible pure state decompositions {*p*_*i*_, |*ψ*_*i*_〉_*AB*_} of *ρ*_*AB*_.

Similarly, the concurrence of assistance (COA) of *ρ*_*AB*_ is defined as^28^


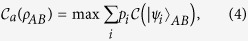


where the maximum is taken over all possible pure state decompositions {*p*_*i*_, |*ψ*_*i*_〉_*AB*_} of *ρ*_*AB*_.

Another well-known quantification of bipartite entanglement is negativity. For any bipartite pure state |*ψ*〉_*AB*_, the negativity 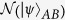 is


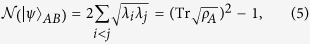


where *ρ*_*A*_ = *tr*_*B*_(|*ψ*〉_*AB*_〈*ψ*|).

For any bipartite state *ρ*_*AB*_ in the Hilbert space 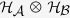 negativity is defined as^29^


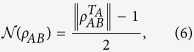


where 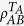 is a partial transposition with respect to the subsystem *A*, 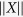 denotes the trace norm of *X*; i.e., 
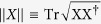
. Negativity is a computable measure of entanglement, which is a convex function of *ρ*_*AB*_. It disappears if, and only if, *ρ*_*AB*_ is separable for the 2 ⊗ 2 and 2 ⊗ 3 systems^30^. For the purposes of this discussion, we use the following definition of negativity:


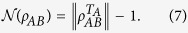


For any maximally entangled state in a two-qubit system, this negativity is equal to 1. CREN gives a perfect discrimination of positive partial transposition-bound entangled states and separable states in any bipartite quantum system^31^,^32^. For any mixed state *ρ*_*AB*_, CREN is defined as


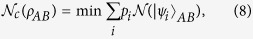


where the minimum is taken over all possible pure state decompositions {*p*_*i*_, |*ψ*_*i*_〉_*AB*_} of *ρ*_*AB*_.

For any mixed state *ρ*_*AB*_, CRENOA is defined as^17^


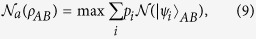


where the maximum is taken over all possible pure state decompositions {*p*_*i*_, |*ψ*_*i*_〉_*AB*_} of *ρ*_*AB*_.

CREN is equivalent to concurrence for any pure state with Schmidt rank-2^17^, and consequently, it follows that for any two-qubit mixed state *ρ*_*AB*_ = ∑_*i*_*p*_*i*_|*ψ*_*i*_〉〈*ψ*_*i*_|:


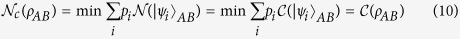


and


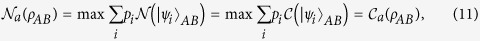


where the minimum and the maximum are taken over all pure state decompositions {*p*_*i*_, |*ψ*_*i*_〉_*AB*_} of *ρ*_*AB*_.

### Monogamy relations of concurrence and negativity

The CKW inequality^8^ was first defined as


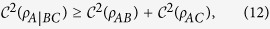


where 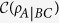 is the concurrence of a three-qubit state *ρ*_*A*|*BC*_ for any bipartite cut of subsystems between *A* and *BC*. Similarly, the dual inequality in terms of COA is as follows^33^:


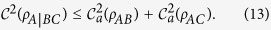


For any pure state 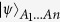 in an *n*-qubit system *A*_1_⊗...⊗*A*_*n*_, where *A*_*i*_ ≅ *C*^2^ for *i* = 1, ..., *n*, a generalisation of the CKW inequality is


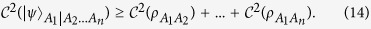


The dual inequality in terms of the COA for *n*-qubit states has the form^17^


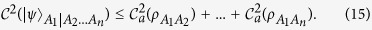


when the rank of the matrix is 2, we have


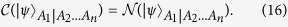


Combining Eq. (10) with Eq. (11), we have


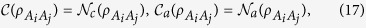


where *i*, *j* ∈ {1, ..., *n*}, *i* ≠ *j*.

For any *n*-qubit pure state 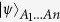, we have


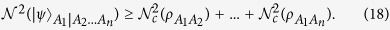


The dual inequality^17^ in terms of CRENOA is as follows:


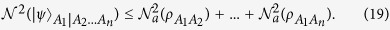


### Monogamy inequalities of CREN

For a 2 ⊗ 2 ⊗ *m* quantum pure state |*ψ*〉_*ABC*_, it has been shown that 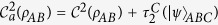
33, where 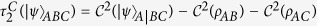 is the three-tangle of concurrence. 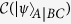 is the concurrence under bipartition *A*|*BC* for pure state |*ψ*〉_*ABC*_. Namely,


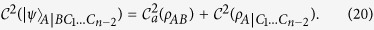


Similarly, considering that CREN is equivalent to concurrence by Eq. (17), we have


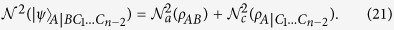


The concurrence is related to the linear entropy of a state^34^


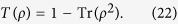


Given a bipartite state *ρ*, *T(ρ*) has the property^35^,


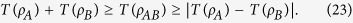


From the definition of pure state concurrence in Eq. (2) together with Eq. (22), we have


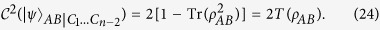


Now, we provide the following theorems:

Theorem 1. For any 2 ⊗ 2 ⊗ 2 tripartite mixed state *ρ*_*ABC*_ we have


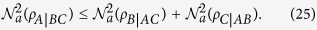


Proof. Let *ρ*_*ABC*_ = ∑_*i*_*p*_*i*_|*ψ*_*i*_〉_*ABC*_〈*ψ*_*i*_| be an optimal decomposition realising 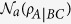; that is,


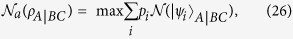


where *ρ*_*BC*_ = Tr_*A*_|*ψ*_*i*_〉_*ABC*_〈*ψ*_*i*_|, *ρ*_*B*_ = Tr_*AC*_|*ψ*_*i*_〉_*ABC*_〈*ψ*_*i*_| and *ρ*_*C*_ = Tr_*AB*_|*ψ*_*i*_〉_*ABC*_〈*ψ*_*i*_|, and we have


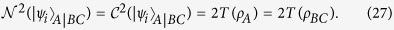


Combining Eq. (23) with Eq. (24), we have


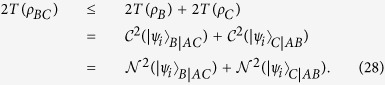


The third equality holds because CREN and concurrence are equal for any rank-2 pure state. Therefore, we obtain


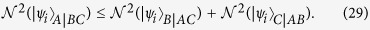


Combining Eq. (26) with Eq. (29), we finally get


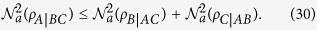


Thus, the proof is completed.

Theorem 1 shows a simple relationship of CRENOA in a tripartite quantum system. The monogamy inequality shows that the entanglement *A*|*BC* cannot be greater than the sum of the entanglement *B*|*AC* and the entanglement *C*|*AB*. Taking an easy example, when considering a three-qubit state, the following equation exists: |*ψ*〉_*ABC*_ = *a*|010〉 + *b*|100〉 where |*a*|^2^ + |*b*|^2^ = 1. Using a simple calculation, the following equation can be obtained: 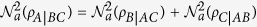 where the state |*ψ*〉_*ABC*_ saturates the monogamy inequality in Eq. (25). Moreover, the iteration of Eq. (25) leads us to the generalized monogamy inequality in multi-qubit quantum systems.

Corollary 1. For any multi-party mixed state 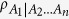 in an *n*-qubit system^36^, the following monogamy inequality exists:


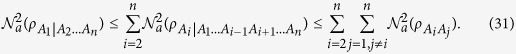


**Figure 1:**
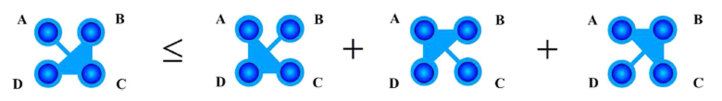
The example shows the reciprocal relation of squared CRENOA in a four-qubit system.

The meaning of the first inequality is clear the bipartite entanglement between 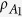 and the other qubits, when taken as a group cannot be greater than the sum of the *n* − 1 individual bipartite entanglements between 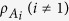 and the other remaining qubits. We now start to consider a four-qubit system. As shown in Fig. 1, the squared CRENOA with respect to the bipartition (*A*|*BCD*) is not greater than the sum of the three squared CRENOAs (the three possible bipartitions are *B*|*ACD*, *C*|*ABD* and *D*|*ABC*).

The meaning of the second inequality is clear the sum of the bipartite entanglements between 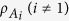 and the other remaining qubits cannot be greater than the sum of the bipartite entanglements 
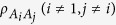
.

Theorem 2. For any *n*-qubit pure state 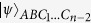, we have


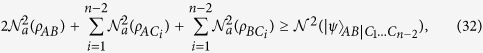


where 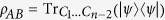, 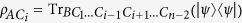 and 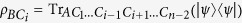.

Proof. From the result of Theorem 1, we find that the generalised monogamy inequality can be easily obtained by using the superposition of states. We now consider 
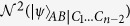
. When the rank of the matrix is 2, we have


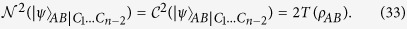


Combining Eq. (23) with Eq. (24), we get the relationship


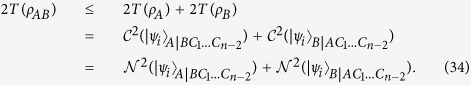


The third equality follows from the fact that CREN and concurrence are equal for any rank-2 pure state.


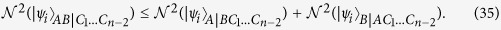


For a mixed state, CRENOA is expressed as 
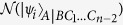
, and we have


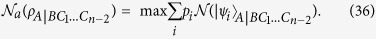


Furthermore, when combining this with Eq. (35), we finally get


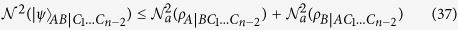


and


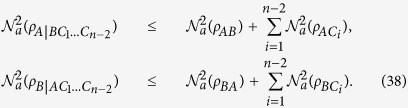


Combining Eq. (37) with Eq. (38), we have Eq. (32). In other words, we give an upper bound about 
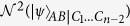
, i.e.,


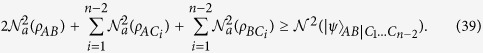


This completes the proof.

Theorem 2 shows that the entanglement between *AB* and the other qubits cannot be greater than the sum of the individual entanglements between *A* and each of the *n* − 1 remaining qubits and the individual entanglements between *B* and each of the *n* − 1 remaining qubits. Theorem 2 provides a polygamy-type upper bound of multi-qubit entanglement between the two-qubit system *AB* and the other (*n* − 2)-qubit system *C*_1_*C*_2_...*C*_*n*−2_ in terms of the squared CRENOA. Especially under partition *AB*, a two-qubit system is different from the previous monogamy inequality. When 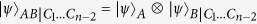, the calculation results in 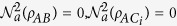. Consequently, the polygamy-type relation is obtained as shown in Eq. (19).

Finally, consider the following four-qubit state: |*ψ*〉_*ABCD*_ = *a*|0100〉 + *b*|0010〉 + *c*|0001〉 where |*a*|^2^ + |*b*|^2^ + |*c*|^2^ = 1. We can easily get the following equations: 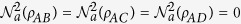 and 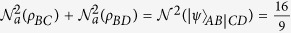. Therefore, the state |*ψ*〉_*ABCD*_ saturates the monogamy inequality in Eq. (32).

Theorem 3. For any *n*-qubit pure state 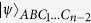,


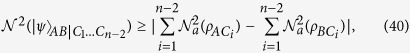


where 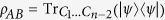, 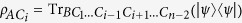 and 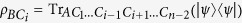.

Proof. We have the following property for linear entropy^35^:


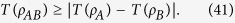


Combining Eq. (24) with Eq. (41), we have


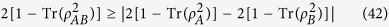


and


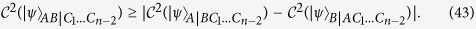


By using the equivalent relation between concurrence and CREN (see Eq. (17)), we have


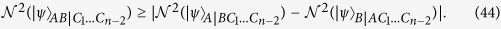


There is a relationship between CREN and CRENOA (see Eq. (21)):


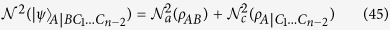



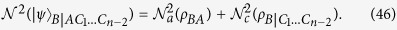


Putting the above two equalities into Eq. (44), we get


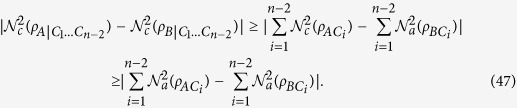


Similar to the above derivation, we give a lower bound about 
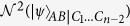
, i.e.,


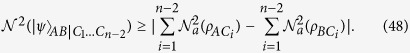


This lower bound is a direct consequence of CREN.

Theorem 3 shows that the entanglement between *AB* and the other qubits cannot be less than the absolute value of the difference between both the individual entanglements between *A* and each of the *n* − 1 remaining qubits and the individual entanglements between *B* and each of the *n* − 1 remaining qubits. Theorem 3 provides a monogamy-type lower bound of multi-qubit entanglement between the two-qubit system *AB* and the other (*n* − 2)-qubit system *C*_1_*C*_2_...*C*_*n*−2_ in terms of the squared CRENOA. When 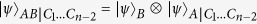, 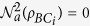, and so we obtain the CWK-type relation in Eq. (18).

Finally, we consider the following four-qubit state |*ψ*〉_*ABCD*_ = *a*|1000〉 + *b*|0010〉 + *c*|0001〉 where |*a*|^2^ + |*b*|^2^ + |*c*|^2^ = 1, from which we can easily obtain the following equations: 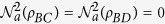 and 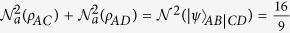. Therefore, the state |*ψ*〉_*ABCD*_ saturates the monogamy inequality in Eq. (40). Therefore, a generalised monogamy inequality using negativity and CRENOA in an *n*-qubit is proposed. These relations also give rise to a type of trade-off in inequalities that is related to the upper and lower bounds of CRENOA.

Remark. It is interesting to note that the properties of CREN are based on the subadditivity of linear entropy. However, negativity violates this subadditivity in general conditions^37^,^38^,^39^.

### Examples

In this section, we use some special states to study generalised monogamy inequalities. First, we consider the (Greenberger-Horne-Zeilinger) GHZ state and W state in Examples 1 and 2. Second, we consider two states in the higher-dimensional system in Examples 3 and 4.

Example 1. For an arbitrary pure GHZ state in an *n*-qubit system:


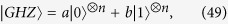


where |*a*|^2^ + |*b*|^2^ = 1. The generalized GHZ state is satisfied with the previous CKW inequality. We will now show that the generalised GHZ state satisfies the generalised monogamy inequalities. We have *ρ*_1_ = *ρ*_2_ = … = *ρ*_*n*_ = *a*^2^|0〉〈0| + *b*^2^|1〉〈1|. It is straightforward to check: 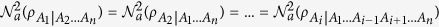

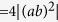
 and 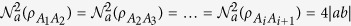, 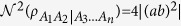. Therefore:


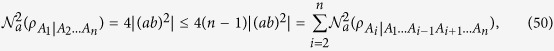



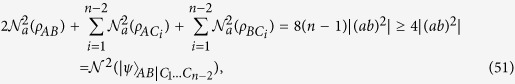



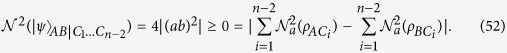


Example 2. For a pure state |*W*〉 in an *n*-qubit system:


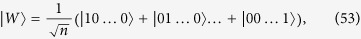


with 
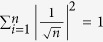
. It is very important to understand the saturation of the previous CKW inequality. Using a simple calculation, we have 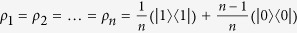. It is straightforward to check: 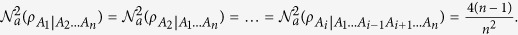 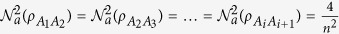, 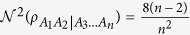. In the same way, we get the following inequalities:


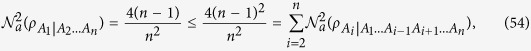



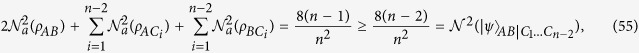



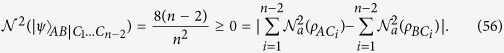


From the above results, we discover that the generalised GHZ state and W state satisfy our inequalities. We further explore the condition of the generalised inequalities in higher-dimensional systems. We consider the following examples:

Example 3. For a pure, totally antisymmetric state |*ψ*_*ABC*_〉 in a 3 ⊗ 3 ⊗ 3 system^40^:


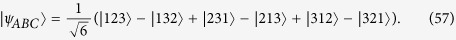


This special quantum state is not satisfied with the previous CKW inequality^41^ but it is established in generalised monogamy inequalities. We can easily obtain 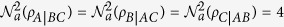 and further obtain the inequalities 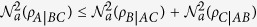. We now explore theorems 2 and 3. First, we have 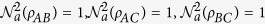 and 
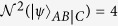
. Therefore, we obtain the following inequalities:


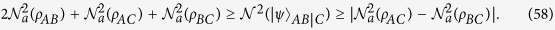


Example 4. The *n*-qudit generalised W-class state in higher-dimensional quantum systems is very useful in quantum information theory^42^. We verify whether the generalised monogamy inequalities hold in higher-dimensional systems using a special example. First, we recall the definition of *n*-qudit generalised W-class state^43^,


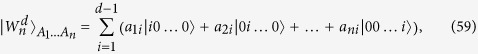


where 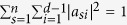.

Let 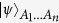 be an *n*-qudit pure state in a superposition of an *n*-qudit generalised W-class state and vacuum; that is,


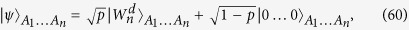


for some 0 ≤ *p* ≤ 1.

For the squared negativity 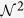 of 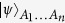 with respect to the bipartition between *A*_1_ and the other qudits, the reduced density matrix 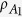 of 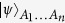 onto subsystem *A*_1_ is obtained as


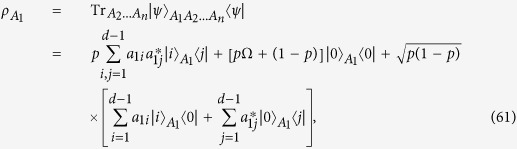


where 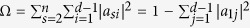.

When considering the  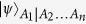 state, we need to obtain the eigenvalue of the matrix by applying the definition of pure state negativity in Eq. (5). Using a simple calculation, we find that the matrix has rank-2 and we have


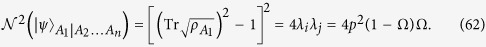


We now consider the case in which *n* = 2. The remaining cases follow analogously. The two-qudit reduced density matrix 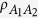 of 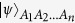 is obtained as


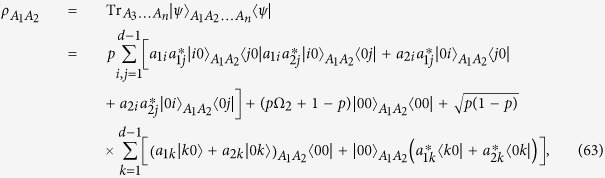


where 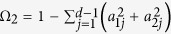. For convenient calculation, we consider two unnormalised states:


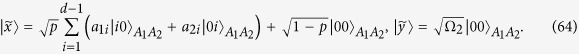


Consequently, 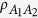 can be represented as 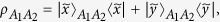 where 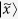 and 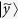 are unnormalised states of the subsystems *A*_1_*A*_2_. By the HJW theorem^44^, any pure-state decomposition 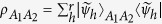, with size *r* > 2 can be obtained by an *r* × *r* unitary matrix *u*_*hl*_ such that


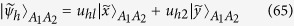


for each *h*, for the normalized state  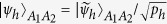  with 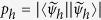.

We apply the definition of mixed state negativity in Eqs (8 and 63), and then we have the two-tangle based on the CREN of 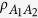 as


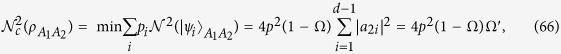


where 
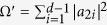
.

From the definition of pure state negativity in Eqs (9 and 63), we have


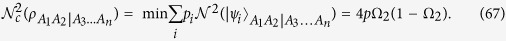


We now try to verify the generalised monogamy inequalities of CREN in an *n*-qudit system. For convenient calculation, we assume that 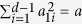, 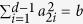, 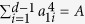, 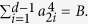


We first consider the generalisation of Theorem 1.


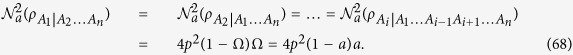


This special quantum state is satisfied with the generalised monogamy inequality in Eq. (25) i.e.,


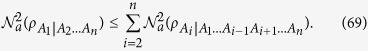


For the generalisation of Theorem 2, the left of Eq. (32) is


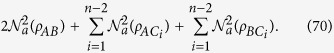


Using Eqs (8 and 62) we can simplify the calculation to


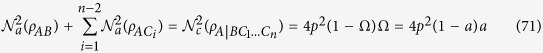


and


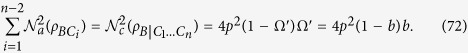


After some calculations, we have


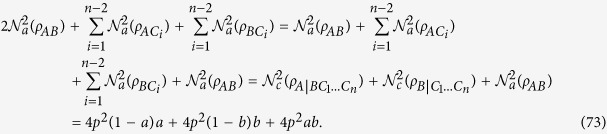


Second, taking Eq. (67) to the right side of Eq. (32), we then have


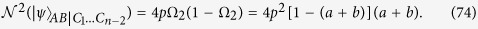


After a straightforward calculation, we obtain


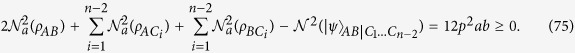


Therefore, this *n*-qudit pure state is satisfied with the generalised monogamy inequality in Eq. (32). In other words, the test of the Theorem 2 has been accomplished. Next, we verify Theorem 3. First, we consider the term CREN from Eq. (40):


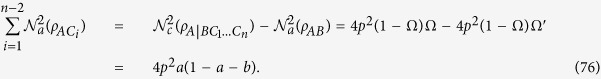


Calculating the absolute value of the difference between Eqs (72 and 76), we obtain


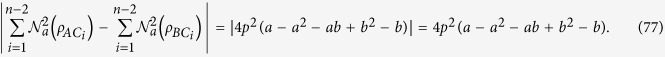


It is easy to check 4*p*^2^ (*a* − *a*^2^ − *ab* + *b*^2^ − *b*) > 0, as


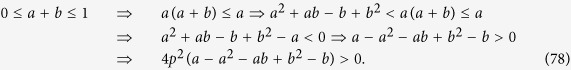


After a straightforward calculation, we have


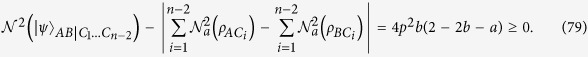


Therefore, this *n*-qudit pure state satisfies the generalised monogamy inequality in Eq. (40). We have now verified the generalised monogamy inequalities. In other words, the generalised monogamy inequality are satisfied with the *n*-qudit pure state for all three of our theorems.

## Conclusions

In this paper, we have used CREN to study different types of monogamy relations. In particular, we have shown that CREN satisfies the generalised monogamy inequalities. We have investigated the CKW-like inequalities and generalised monogamy inequalities. Furthermore, the generalised monogamy inequalities related to CREN and CRENOA were obtained by *n*-qubit states. These relations also give rise to a type of trade-off in inequalities that is related to the upper and lower bounds of CRENOA. Finally, we have shown that the partially coherent superposition of the generalised W-class state and vacuum extensions of CREN satisfies the generalised monogamy inequalities. We believe that the generalised monogamy inequalities can be useful in quantum information theory. This paper was based on the linear entropy. To continue this work, we will study the nature of other entropy further in the future work. We hope that our work will be useful to the quantum physics.

## Additional Information

**How to cite this article**: Tian, T. *et al*. Generalised monogamy relation of convex-roof extended negativity in multi-level systems. *Sci. Rep.* **6**, 36700; doi: 10.1038/srep36700 (2016).

**Publisher’s note:** Springer Nature remains neutral with regard to jurisdictional claims in published maps and institutional affiliations.
